# Prediction of COVID-19 Data Using Hybrid Modeling Approaches

**DOI:** 10.3389/fpubh.2022.923978

**Published:** 2022-07-22

**Authors:** Weiping Zhao, Yunpeng Sun, Ying Li, Weimin Guan

**Affiliations:** ^1^School of Asian Languages, Zhejiang Yuexiu University of Foreign Language, Shaoxing, China; ^2^School of Economics, Tianjin University of Commerce, Tianjin, China

**Keywords:** hybrid modeling approach, ARIMA models, SIR and SIER models, COVID-19, Pakistan

## Abstract

A major emphasis is the dissemination of COVID-19 across the country's many regions and provinces. Using the present COVID-19 pandemic as a guide, the researchers suggest a hybrid model architecture for analyzing and optimizing COVID-19 data during the complete country. The analysis of COVID-19's exploration and death rate uses an ARIMA model with susceptible-infectious-removed and susceptible-exposed-infectious-removed (SEIR) models. The logistic model's failure to forecast the number of confirmed diagnoses and the snags of the SEIR model's too many tuning parameters are both addressed by a hybrid model method. Logistic regression (LR), Autoregressive Integrated Moving Average Model (ARIMA), support vector regression (SVR), multilayer perceptron (MLP), Recurrent Neural Networks (RNN), Gate Recurrent Unit (GRU), and long short-term memory (LSTM) are utilized for the same purpose. Root mean square error, mean absolute error, and mean absolute percentage error are used to show these models. New COVID-19 cases, the number of quarantines, mortality rates, and the deployment of public self-protection measures to reduce the epidemic are all outlined in the study's findings. Government officials can use the findings to guide future illness prevention and control choices.

## Introduction

The economy of Pakistan, home to over 197 million people, is rated as being of moderate to medium complexity. Since the first case in Pakistan in February 2020, the country has been on high alert. Government officials and healthcare professionals recommend preventive steps to limit the disease's spread. There are four provinces and three regions in Pakistan: Punjab, Sindh, Khyber Pakhtunkhwa (KPK), Baluchistan, Gilgit-Baltistan, and Azad Jammu & Kashmir (AJK) (1). The first two instances of COVID-19 in Pakistan sounded the warning bell on February 26, 2020 (2). Determine the effects of social isolation remains a pressing issue, and the worldwide health catastrophe of COVID-19 has highlighted the importance of research and scientific progress in the face of this global health epidemic. In addition to the evident deaths and illnesses, the epidemic has caused emotional anguish and stress in the residents, who have been under lockdown and quarantine for the past few months (3). LR, ARIMA, SVR, MLP, RNN, GRU, and long short-term memory (LSTM) are utilized for the same purpose. The root mean square error (RMSE), mean absolute error (MAE), and mean absolute percentage error (MAPE) are used to show these models, and there are a variety of mathematical approaches to the susceptible–exposed–infectious–removed (SEIR) model (4). Health facilities, transportation, and strong economic activity or densely occupied migratory workers might all affect the relevance of infectiousness in a district's population mobility (5). The logistic growth model (6), stochastic susceptible–infectious–removed (SIR) model (7), and SEIR model are used to investigate and predict the future trends of COVID-19 (8). According to Pakistan's COVID-19 statistics, men accounted for 74% of cases in June 2020. However, this might be due to a bias in testing (9). The study consists of five sections. The first section describes the introduction, and section literature review consists of the literature review to describe the supporting references. Section research methodology determined the methodology and research design. Section results and discussions explains the results in the figures and tables, and section conclusions concludes with the results and analysis of the study.

## Literature Review

Infections of the respiratory tract are the most frequent globally, resulting in a high mortality rate and a substantial financial load on healthcare systems (10). Mobile workforce prioritization reduces the spread of diseases by 24.14% compared to other situations. Quezon City's population is best protected by preferring the elderly (439%) over other alternatives (11). The COVID-19 virus has been implicated in an epidemic of atypical pneumonia (12), and the virus's human-to-human transmissibility has been documented domestically and globally (13). Pakistan has seen a comparatively low number of infections and fatalities from the COVID-19 epidemic. There are 585,435 cases and 13,076 fatalities in the country as of March 4, 2021 (14). The mathematical modeling and forecasting of epidemics have been resurrected after COVID-19's outbreak are developing new prediction algorithms to track the spread of the disease and foretell its future course (15, 16). However, quarantine is more effective at restricting the transmission of the virus, a control plan that maximizes and is likely to be successful. For release from quarantine, continuous, and stringent compliance with prescribed social and health-enhancing activities is required (17). Since no vaccine has been created for infectious diseases, such as COVID-19, vaccinations cannot be used to treat them. A limited supply of vaccines controlling the spread of COVID-19 requires a focus on early detection and prevention (18). COVID-19 many prediction models are currently available, including the logistic regression analysis LSTM, ARIMA, and TBATS models (19). Alzahrani et al. (20) described that the ARIMA prediction model is used to propagate pandemic outbreaks in Saudi Arabia. COVID-19 lockdown effectiveness is examined using the age-structured variant of the SEIR-type for pandemics (21, 22). The COVID-19 epidemic dynamics are predicted using a modified SEIR compartmental model (23). The dynamic process of epidemic spread is described using a simple SEIR model developed (24). The average age of the people of Quezon City is significantly lower than that of the most vulnerable demographic (22). COVID-19's spread may be predicted using SEIR's mathematical modeling. The SEIR model is utilized to build the model (25). The model study employs the generation matrix approach (26) to get the fundamental reproduction number and the global stability for COVID-19 spreading by incorporating vaccination and isolation variables as model parameters. The number of COVID-19 cases in Pakistan is simulated using secondary data (27). Compared to the Q-SEIR model, the infection rates predicted by the age-stratified probability are significantly lower. Quarantine delays peaks for exposed and infected groups and “flattens” the curve or lowers each compartment's projected value (28). The effectiveness of various actions following the outbreak may be evaluated (29), which appears to be a daunting problem for general statistical approaches. As a general rule, the SIR paradigm is commonly used to describe the progression of an illness from one person to another through three distinct but incompatible stages (15). The long-term dependencies in huge sequences of hundreds or thousands of steps are the fundamental restriction of RNN learning. LSTM networks can overcome these restrictions (30). Precautions include immunizing pupils first, mandating mask use, and maintaining a physical distance of at least 25 feet between students and teachers (31). Chinese forecasters employed LSTM for COVID-19 forecasting and found it more accurate than the dynamic SEIR model (32). The system uses a SIR model and an LSTM to depict the current state of the pandemic and estimate its course into the future with reasonable accuracy (33). The RMSE calculates the difference between the actual and fitted data, while the R shows the relationship between the model's output and the actual data (34). LSTM networks, polynomial neural networks, and neural networks may all be used to anticipate COVID-19 situations (35).

## Research Methodology

### Data Collection

The research data is collected from March 1, 2020 to September 30, 2020, and the number of confirmed COVID-19 cases is utilized to conduct the investigation using SIR and SEIR models. The COVID-19 cases data of Pakistan is collected from the WHO https://www.who.int/.com. The data relating to several total tests confirmed cases, critical cases, death cases, quarantine, and fully vaccinated persons in all provinces in Punjab, Sindh, Baluchistan, KPK, and Jammu & Kashmir is collected from the National Conference on Citizenship (NCOC) Govt. of Pakistan website.”

### Research Design

Rao et al. (36) propose a non-linear incidence rate and the recovery rate in the dimensional model discussed. Hospital bed-population ratio “*a*_1_> 0” and contaminated “*I*” are both factors that affect the patient's ability to recover; according to the healing rate (37).


(1) $$\begin{array}{l} b=b_{0}+\frac{\left(b_{1}\;-\;b_{0}\right)a_{1}}{1\;-\;a_{1}} \end{array}$$

The maximum and minimum per capita recovery rates are represented by the parameters *a*_1_ and *a*_0_ can use the function to generalize a nonlinear incidence rate.


(2) $$\begin{array}{l} \text{f(S, I)}=\frac{\alpha_{1}\;SI}{b_{1}\;+\;b_{2}S\;+\;b_{3}I} \end{array}$$

Thus, the system of differential equations is given by


(3) $$\begin{array}{l} \frac{dS}{dt}=\left(1\;-q\right)\;\text{a}-\sigma_{1}\text{S}-\text{f}\left(S,\;I\right)+\gamma \text{R}, \end{array}$$

(4) $$\begin{array}{l} \frac{dI}{dt}=\text{f}\left(S,\;I\right)+\left(\sigma_{2}\;+b\right)I, \end{array}$$

(5) $$\begin{array}{l} \frac{dR}{dt}=\text{qa}-\left(\sigma_{2}\;+\;\gamma \right)\text{R}+bI, \end{array}$$

*S* (*t*) is the susceptible population, *I* (*t*) is the population that is infected, and *R* (*t*) is the population that has recovered, so that *N* = *S* + *I* + *R*.

### Logistic and SEIR Hybrid Model

As a result, this model can only forecast cumulative infections but not the present number of confirmed cases. The SEIR model can simulate population shifts resulting from infectious disease outbreaks. According to Nawaz et al. (38), if the number of confirmed COVID-19 diagnoses each day (*I*) is projected, the number of healthy persons (*S*), exposed people (*E*), and infected people (*I*) need to be computed. It is also necessary to examine the number of recovered persons (*R*), the infection rate of healthy people becoming latent, the infection rate of exposed people getting infected, and the recovery rate of diseased people. The rationale behind the SEIR and the logistic model is that the logistic model's parameters are the initial number of infections P_0_, the cumulative number of infections *K*, and the infection rate (*r*), which may be automatically produced after dataset training. We may utilize (*r*) in the logistic model to initialize in the SEIR model. The SEIR model's starting numbers of infected people (*I*) and exposed people (*E*) are set to zero when *P*_0_ is used in the logistic model. When calculating the number of rehabilitated persons (*R*), the first day's total and the number of dead people in the data are used as a starting point. It can start SEIR model (*S*) by multiplying the Logistic model (*K*) by a coefficient larger than (1) if the starting number of healthy persons within a specified range is similar to or more than the ultimate cumulative number of illnesses. It may make reasonably simple adjustments to the infection and recovery rates of those exposed and those who have been affected. The accuracy of the predictions will rise. The algorithm and implementation of the Logistic SEIR model.

Logistic function as defined in Equation 1, logistic function (*t, K*, *P*_0_, *r*):


(1) $$\begin{array}{l} Return=kQ_{0}e^{rt}/\left[\text{k}+Q_{0}\left(e^{rt}-1\right)\right] \end{array}$$

Define the SEIR model function according to Equations 2–5:

*diff_SEIR (Y* = *[S, E, I, R])*:


(2) $$\begin{array}{l} \text{X(0)}=-\beta \times \text{Y(0)}\times \text{Y(2)} \end{array}$$

(3) $$\begin{array}{l} \text{X(i)}=\beta \times \text{Y(0)}\times \text{Y(2)}-\text{Y(1)}\times \text{b} \end{array}$$

(4) $$\begin{array}{l} \text{X(ii)}=b\times \text{Y(1)}\gamma \times \;\text{Y(2)} \end{array}$$

(5) $$\begin{array}{l} \text{X(iii)}=\gamma \times \;\text{Y(2)} \end{array}$$

Reappearance Y

(1) The initial number of infected persons (*Q*_0_) and the ultimate cumulative number of infected people (*k*) may be determined using the nonlinear least-squares approach to fit the logistic function on the training set (*r*).
(2) In this step, use the previously collected parameters to initialize the SEIR model parameters partially and then initialize the remaining parameters.
(3) The SEIR model differential equation is solved in Matlab, and the projected number of confirmed patients is obtained.

From 7 to 4, the number and complexity of setting a single SEIR model parameter are considerably reduced by the hybrid model suggested in this article. You can get better prediction results by fine-tuning the parameters within a narrower range.

## RMSE and MAE

The SEIR individual's prediction is not good enough. Seven parameters need to be debugged repeatedly to get a decent prediction effect from the SEIR model, which is a time-consuming and hard operation. A quantitative study of the model's prediction effect uses the RMSE and MAE.


(6) $$\begin{array}{l} RMSE=\sqrt{\frac{1}{M}\Sigma_{T=1}^{M}{\left(z_{t}\;-\;q_{t}\right)}^{2}} \end{array}$$

(7) $$\begin{array}{l} MAE=\frac{\overset{m}{\underset{j=1}{\sum }}|x_{j}\;-\;y_{j}|}{m} \end{array}$$

**Figure 1:**
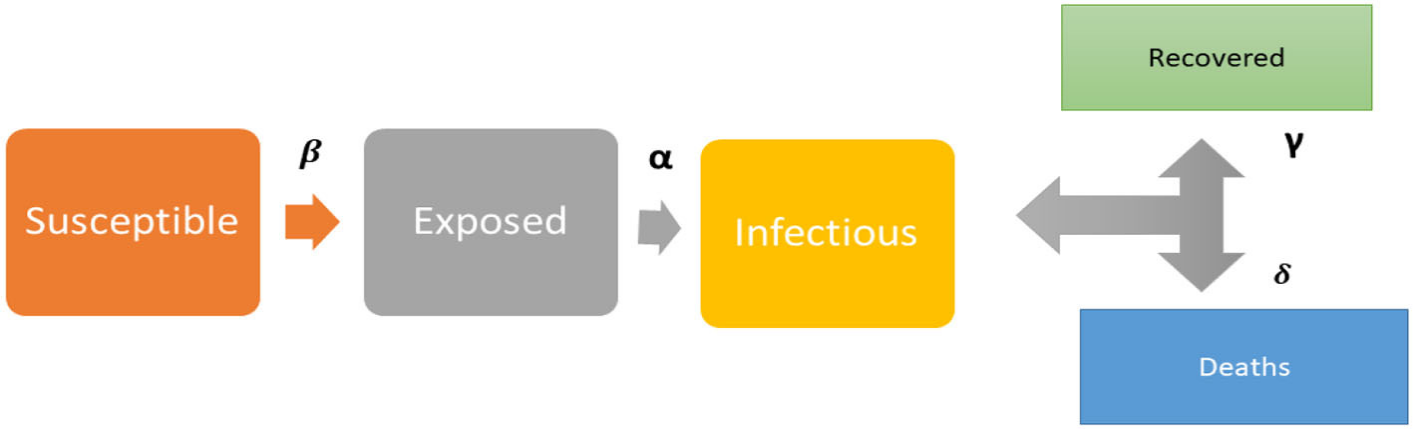
Conceptual framework of the SIER model.

Where *q*_*t*_ and *z*_*t*_ represent the *t*^*th*^ predicted and true values, *M* is the total number of samples for which a predicted, and true values exist. No matter how one looks at the RMSE model provided in this study, it appears to have certain advantages over other models. Figure 1 shows the model's coefficients.

### Algorithmic Demonstration

#### Algorithmic Representation of the Method

**Step-1**: **Start**

**Input**: N data sets

**Step-2: Processing**: Data Cleaning

**Tuning of Parameter**: Based on Logistic growth

**Step-3: Construction of Model:**

Establish a prediction model based on the SIER model.

**Step-4: Validation**:

**Step-5: The parameters are appropriate**

**If Yes - - - Obtain Forecast Result**

**If No - - - Repeat step 2-5**

According to the NCDC, the first incidence of COVID-19 will be reported in January 2020.

Three incidents occurred in February and are consistent throughout the month.

## Results and Discussions

### Hybrid Model-Analysis Results

The hybrid model's SIR and SEIR have been used to study and predict the spread of COVID-19 disease in this review. We used LR, ARIMA, SVR, MLP, RNN, GRU, and LSTM for the same reason. These models are evaluated using RMSE, MAE, and MAPE for their display. From March 2020 to September 2020, the number of confirmed COVID-19 cases is utilized to conduct the investigation using SIR and SEIR models. Table 1 presents the results for each of the study areas.

**Table 1:** Validation matric for COVID-19 confirm case (actual vs. predicted) without temperature.

**Localities**	**Methods**	**RMSE**	**MAE**	**MAPE**
Punjab	ARIMA	20.176	407.102	0.025101
	LR	19.49	380.011	0.023831
	SVR	18.17	330.19	0.026613
	MLP	17.35	301.181	0.020162
	RNN	14.5	210.451	0.019541
	GRU	13.44	180.178	0.01861
	LSTM	12.57	141.91	0.018111
Sindh	ARIMA	20.176	405.102	0.026101
	LR	20.49	380.011	0.028831
	SVR	19.17	330.19	0.027613
	MLP	18.35	307.181	0.020162
	RNN	15.53	210.451	0.019541
	GRU	14.41	180.178	0.01871
	LSTM	12.15	155.91	0.018511
KPK	ARIMA	20.76	409.102	0.027101
	LR	19.49	380.011	0.023831
	SVR	18.72	330.19	0.026613
	MLP	17.53	301.181	0.020162
	RNN	15.5	217.451	0.019541
	GRU	14.44	176.781	0.01891
	LSTM	13.51	146.91	0.017911
Baluchistan	ARIMA	20.176	407.102	0.027101
	LR	19.49	395.011	0.026131
	SVR	18.17	331.19	0.025613
	MLP	17.35	301.181	0.020162
	RNN	14.5	210.451	0.019541
	GRU	13.44	180.178	0.01891
	LSTM	11.65	135.91	0.017911
Azad Jammu	ARIMA	19.1	407.102	0.025101
& Kashmir	LR	18.49	380.011	0.024831
	SVR	17.11	330.19	0.026713
	MLP	16.35	305.181	0.0199162
	RNN	13.51	208.451	0.018941
	GRU	12.44	181.178	0.01881
	LSTM	11.57	138.91	0.018011

The limit LSTM model outperformed all of our other recognized models in performance. In addition, the model's productivity in the AJK district is higher than in other regions. According to information provided by Pakistan's Public Order and Activity Center (NCOC) and used in the evaluation of execution, RMSE for the SEIR model results is expected to have a high value in all regions of Pakistan up to and including the year 2020. The RMSE of the two models is approximately multiple times more than MAPE and MAE, which further confirms our crossover model's predominance. Compared to the SEIR model, three borders should be effectively defined, and more information is anticipated to dissect and introduce the boundaries in the half and half model. Longer-term expectations need to grow the recovery rate more because of quantitative examinations by SIR and SEIR models of RMSE and the recommended model the plague develops. The SEIR model then registers Punjab's *R*_0_ value, greater than that of other regions. Taking a look at the trend is almost guaranteed that the number of instances will increase. Medical professionals, healthcare workers, and others who provide basic support must be protected by established clinical standards. Table 2 contains the starting values utilized in the hybrid SEIR model for calculation purposes and the source data for each region of interest in this study.

**Table 2:** The results of SEIRD parameters.

**Parameters**	**Initial Values used in this study**	**Source**
	***Punjab; Sindh; KPK; Baluchistan; AJK***	
Susceptible (S)	S = 33,000,000; 29,000,000; 27,000,000; 23,000,000; 21,000,000	
	S rate = 33,000,000/64000,000	
	= 0.5156; 0.4531; 0.4218; 0.359375; 0.328125	
Exposed (E)	E = 99,219	
	E ratio = 99,219/64,000,000	https://www.undp.org.com
	= 0.001550; 0.001571; 0.001610; 0.00154; 0.001510	
Infected (I)	I = 2114	https://www.undp.org.com
	I ratio = 0.00003303	
Recovered (R)	R = 95,421; 13,600; 130137; 35,971; 15,916	https://www.undp.org.com
	R ratio = 95,421/64,000,000	
	R = 0.00149; 0.0002125; 0.002033; 0.0005620; 0.0002486	
Deaths (D)	D = number of deaths due to COVID-19	
	= 2231; 2517; 1263; 148; 182	

As a result of running the SEIR model, researchers can understand how quickly the virus might spread throughout Pakistan. The results of this simulation are extremely useful in strengthening the strategic capacity to protect Pakistan's provinces against COVID-19 conditions. Changes in parameters such as susceptible (*S*), exposed (*E*), infected (*I*), recovered (*R*), and mortality rates affect the hybrid model (*D*). Adding to the complexity is the requirement to use extra data to establish the starting values of the various SEIR model parameters. According to the current statistics, the estimated peak number of infected persons is higher in Punjab than in any other province. At the same time, the recovery (*R*) and death (*D*) rates are likewise higher in Punjab. According to statistics from the number of COVID-19 cases analyzed, the COVID-19 spreading model is a SEIRV model in the provinces of Pakistan (Punjab, Sindh, Baluchistan, KPK, and AJK). The study's findings may model the transmission of the COVID-19 virus throughout the country by taking into account vaccination and isolation periods in the COVID-19 population.

### SEIR Intervention of Vaccination

The model is a development of SEIR that incorporates vaccination as an additional intervention. The suggested model uses the predictors listed in the methodological section's parameter table. The structural assumptions of a compartmental epidemiological model have the biggest impact on the results. The SEIRV model aims to evaluate the influence of vaccination on an ongoing pandemic, such as the present COVID-19 one, and examine “international and optimum” vaccine distribution options SEIRV models. The SEIRV model's mean delay coefficient peak values are shown in Table 3.

**Table 3:** The results of SEIRV coefficients and portrayals.

**Coefficients**	**Initial values used in this study**	**Source**
β = *Coefficients of infection*	β = β_0_*k	(39)
	β = *coefficient of infection rate*	
	K = frequency of exposure	
	β_0_ = *probability of infection per*	(40)
	*espoure*	
	β_1_ = 6.31	
	β_2_ = 1.11	
γ= Coefficient of	γ= 1/*T*_*i*_	
Migration rate	*T*_*i*_ = *average recovery time*	
	*T*_*i*_ = 14 *days*	
	γ= 1/14 = 0.071	(13)
δ= Coefficient of Latency	δ_*e*_=average value of Latency	
	=5.1 days	
	=1/5.1	
	=0.196	

The study's strategic mixture model fits well with the information gathered from NCOC and various data sets. Furthermore, this investigation examined the impact of openness and its level on each location. Table 4 records the MAE and RMSE for the mixed-race SIERV model affirmed cases. Even though the available information on COVID-19 is extremely limited, the SIERV model performed well. The RMAE's benefits for recovered and confirmed cases are always <5%, well below the margin of error for confusion. In July and August of 2020, when the entire country is put on lockdown due to passing cases. However, the model achieves an RMAE of just 2.2% on average.

**Table 4:** The MAE and RMAE of the hybrid models.

**Localities**	**Methods**	
	**Logistic hybrid model**	**SEIRV model**
**Punjab**		
MAE	0.89	2.51
RMSE	2.72	30.71
**Sindh**		
MAE	0.91	3.53
RMSE	3.86	34.66
**KPK**		
MAE	0.84	2.23
RMSE	3.11	28.47
**Baluchistan**		
MAE	0.78	2.27
RMSE	2.31	30.17
**KPK**		
MAE	0.719	2.11
RMSE	2.29	24.56

As far as the number of cases and deaths are concerned, the COVID-19 in Pakistan has been rather limited. Five hundred eighty-five thousand four hundred thirty-five cases and 13,076 deaths were recorded throughout the country on March 4, 2021. It has been determined that Sindh, southern Punjab, and KPK are the areas with the highest risk of exposure to COVID-19, based on population density and proximity to family members and access to water, sanitation, and cleanliness. Comparatively, Pakistan has an extraordinarily low number of people afflicted by the COVID-19 and a low number of deaths, especially compared to developed nations like the United States and Germany.

It is not until the crossover arrangement has been selected that the final mixture model is attempted with the last informative index separated from the beginning. Table 5 provides a variety of error estimates for this configuration: Use the MAE for quantitative research to understand the model's influence on expectations better. The SEIR model can simulate the changes in the number of various populations during the spread of infectious diseases. The values show the parameters. In addition, the infection rate β of healthy people becoming latent, the infection rate of exposed people becoming infected α, and the recovery rate of infected people becoming recovered γ need to be considered. Table 6 demonstrates that Pakistan's populous strongly influences the spread of COVID-19 **α** and **γ** parameter impact. From the perspective of the prediction curve and the error, the influence of different initial values of the conversion rate **α** from the exposed person to the infected person on the curve prediction effect is studied. Thus, a total of seven parameters are adjusted.

**Table 5:** The results of all provinces of population size.

**Regions**	**Rate**	**MAE**	**RMSE**
Punjab	K	1.58	12.531
	1.15*K	0.96	8.137
	1.30*K	1.15	3.72
	1.45*K	1.51	5.435
	1.60*K	1.57	6.451
Sindh	K	1.58	11.153
	1.15*K	1.17	8.731
	1.30*K	2.15	4.72
	1.45*K	3.53	4.735
	1.60*K	3.87	5.415
Baluchistan	K	1.66	10.531
	1.15*K	1.11	7.137
	1.30*K	2.15	5.72
	1.45*K	3.53	5.253
	1.60*K	3.71	6.135
KPK	K	1.37	9.531
	1.15*K	1.01	7.107
	1.30*K	1.15	3.52
	1.45*K	1.51	5.351
	1.60*K	1.57	6.401
AJK	K	1.28	8.131
	1.15*K	0.91	3.137
	1.30*K	1.01	2.112
	1.45*K	1.23	2.435
	1.60*K	1.37	3.451

**Table 6:** Determination of the impact of seven parameters.

**Region**	**α**	**γ**
	**1 1/5 1/10 1/15**	**1/10 1/14 1/18 1/20**
**Punjab**		
MAE	1.45 0.94 1.11 1.38	1.53 1.04 0.91 1.05
RMSE	11.07 3.91 7.65 7.871	9.51 6.04 3.41 4.11
**Sindh**		
MAE	1.31 1.27 1.21 1.35	1.61 1.14 0.97 1.11
RMSE	13.11 3.73 7.28 7.371	8.53 5.84 3.57 5.23
**KPK**		
MAE	1.43 0.94 1.11 1.45	1.57 1.21 0.87 0.99
RMSE	9.37 4.10 6.18 7.717	7.35 5.04 4.91 5.91
**Baluchistan**		
MAE	1.37 1.04 1.17 1.39	1.46 1.24 0.93 1.01
RMSE	9.07 4.21 5.81 6.671	7.55 6.11 3.87 6.01
**AJK**		
MAE	1.13 0.74 0.99 1.21	1.33 1.14 0.74 0.81
RMSE	8.71 2.52 6.56 5.713	6.31 4.41 3.91 4.61

**Figure 2:**
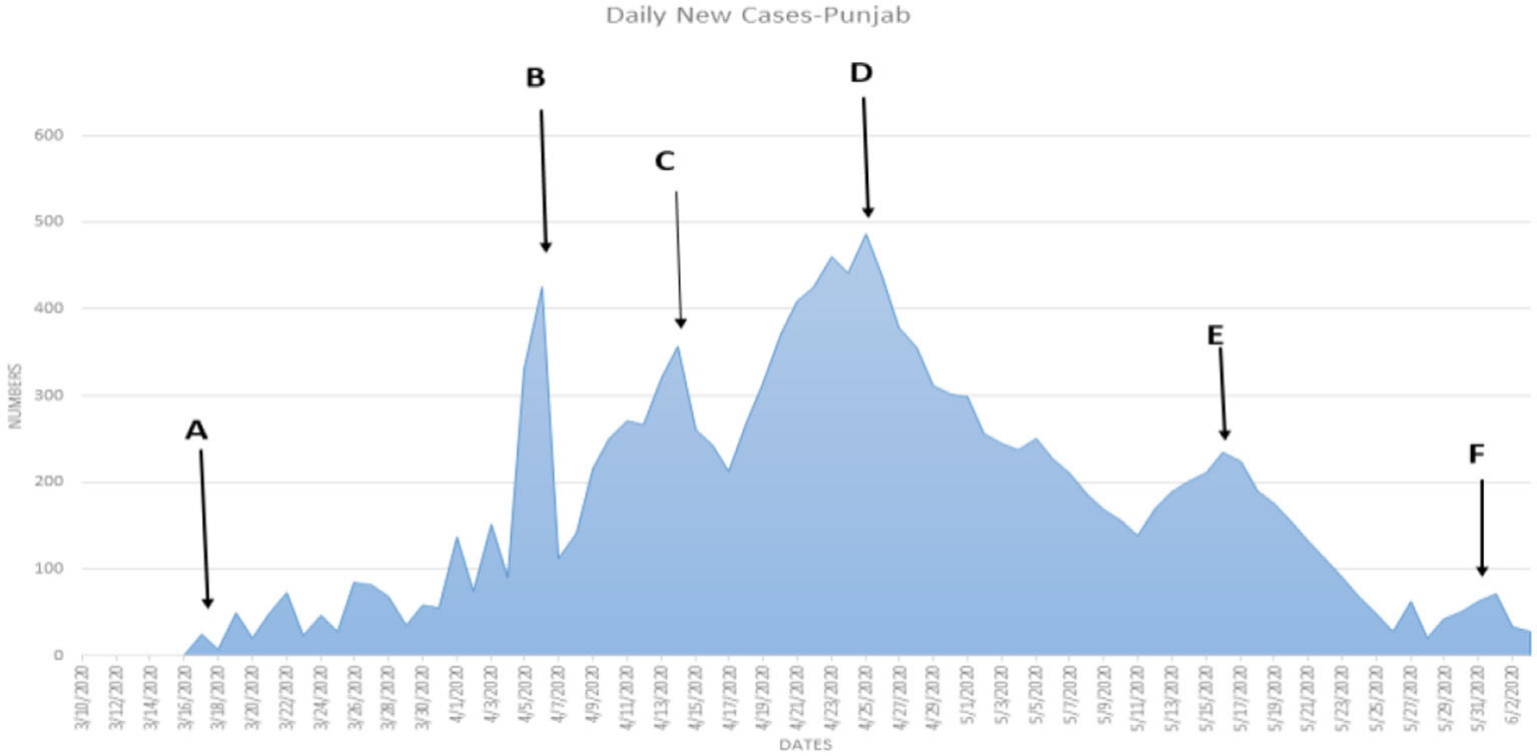
**(A–F)** The number of cases in Punjab.

**Figure 3:**
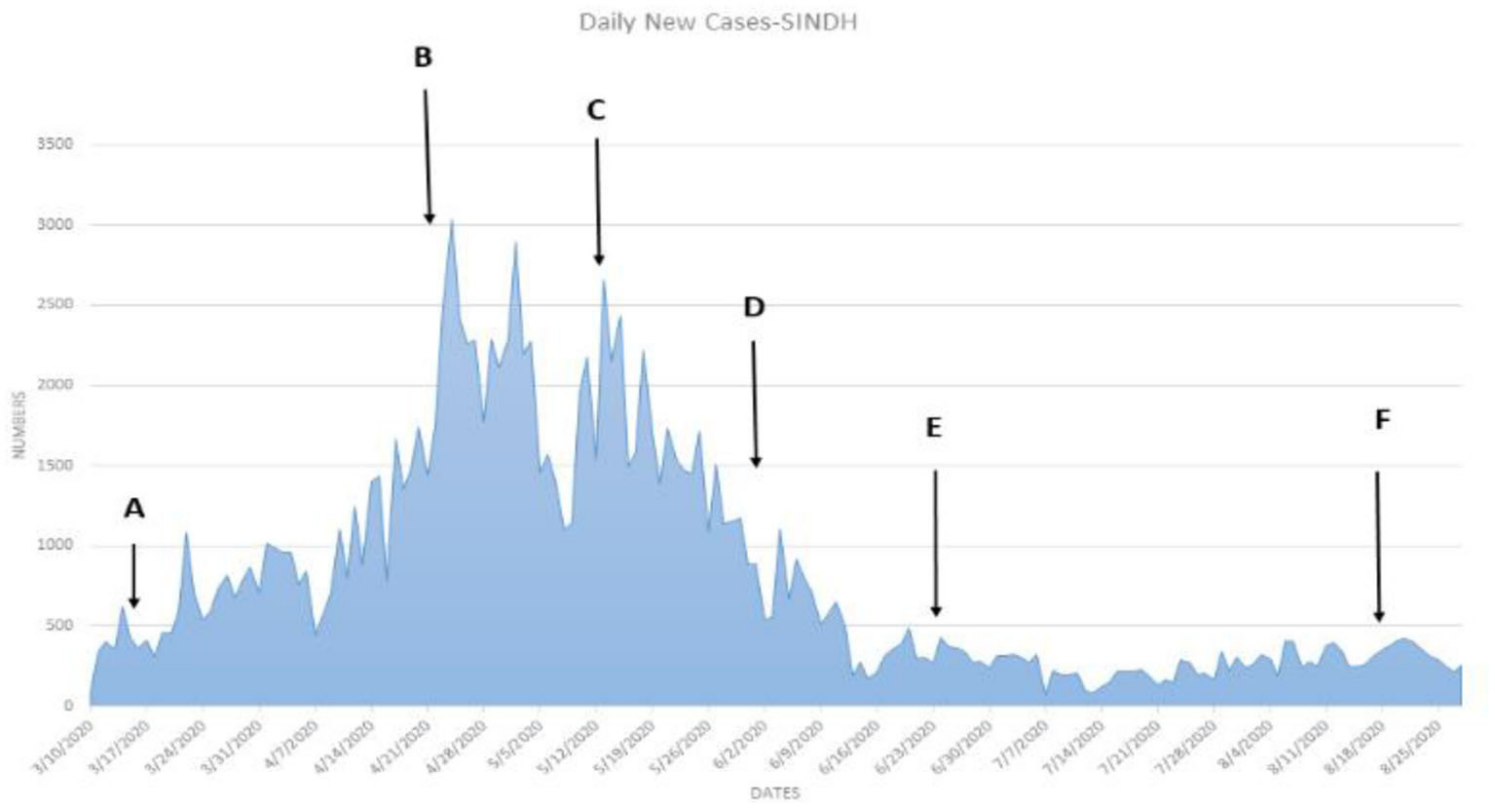
**(A–F)** The number of cases in Sindh.

**Figure 4:**
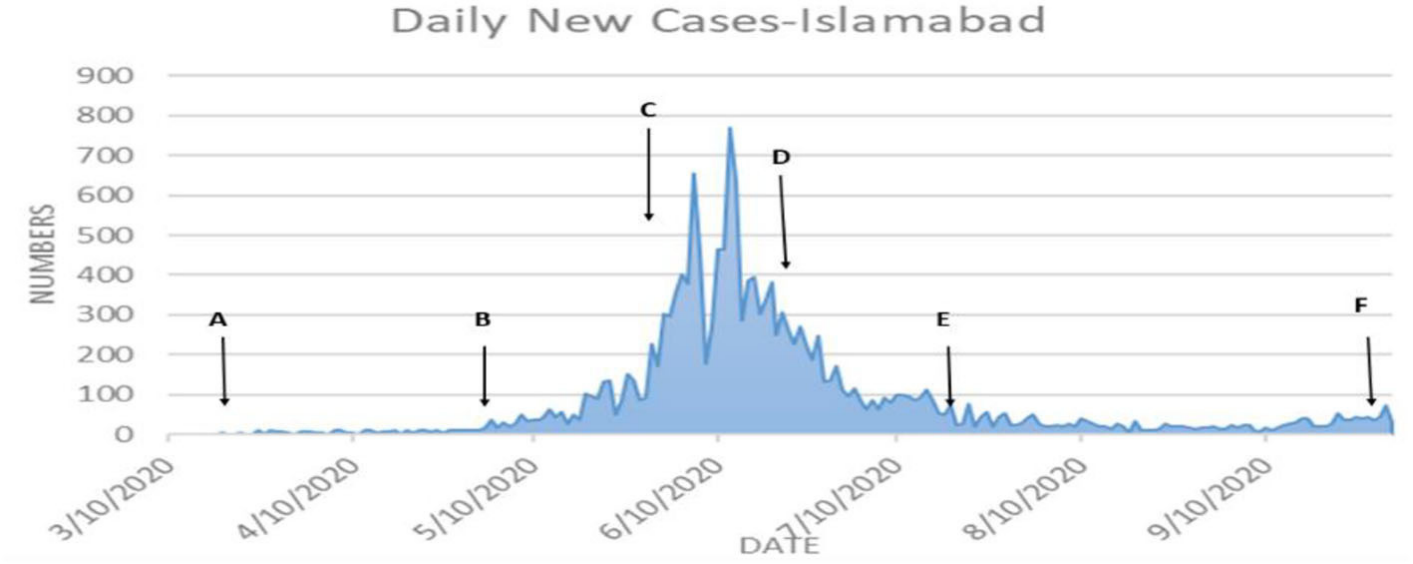
**(A–F)** The number of cases in Islamabad.

**Figure 5:**
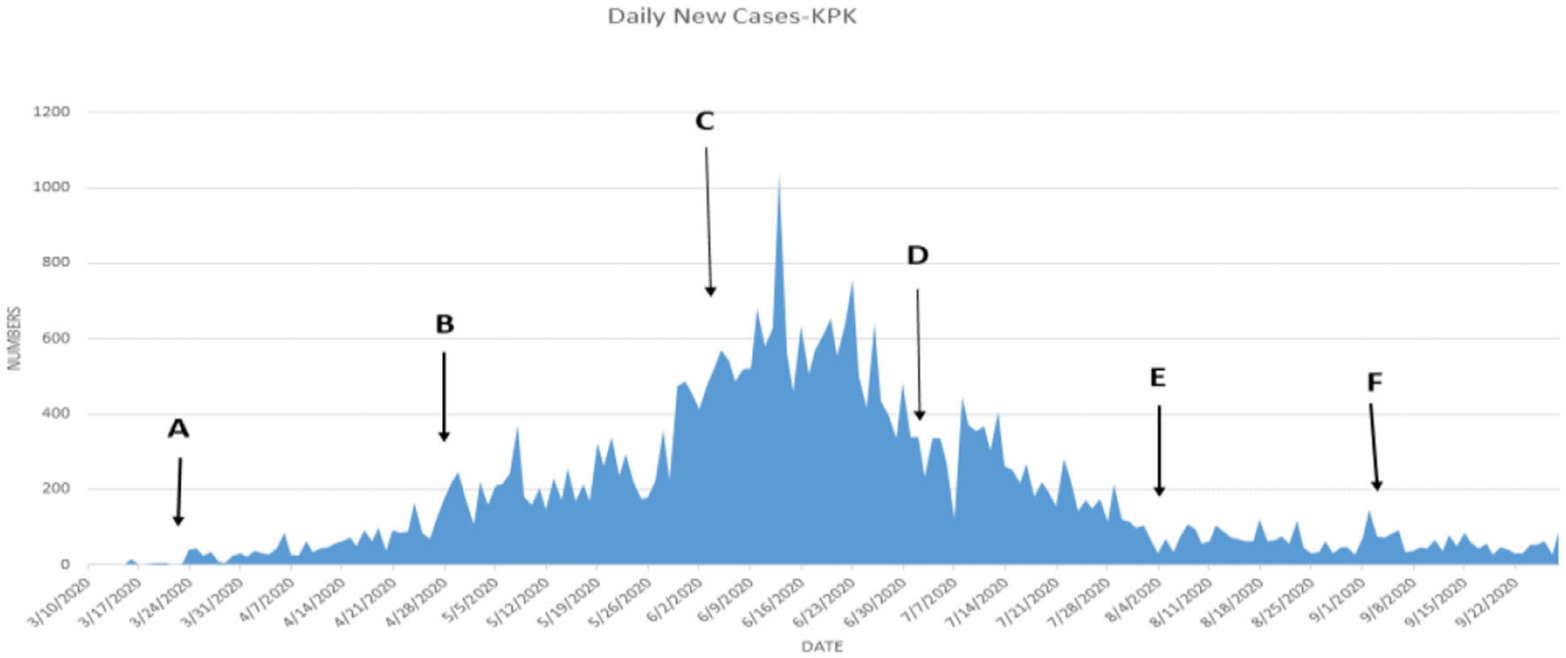
**(A–F)** The number of cases in KPK.

**Figure 6:**
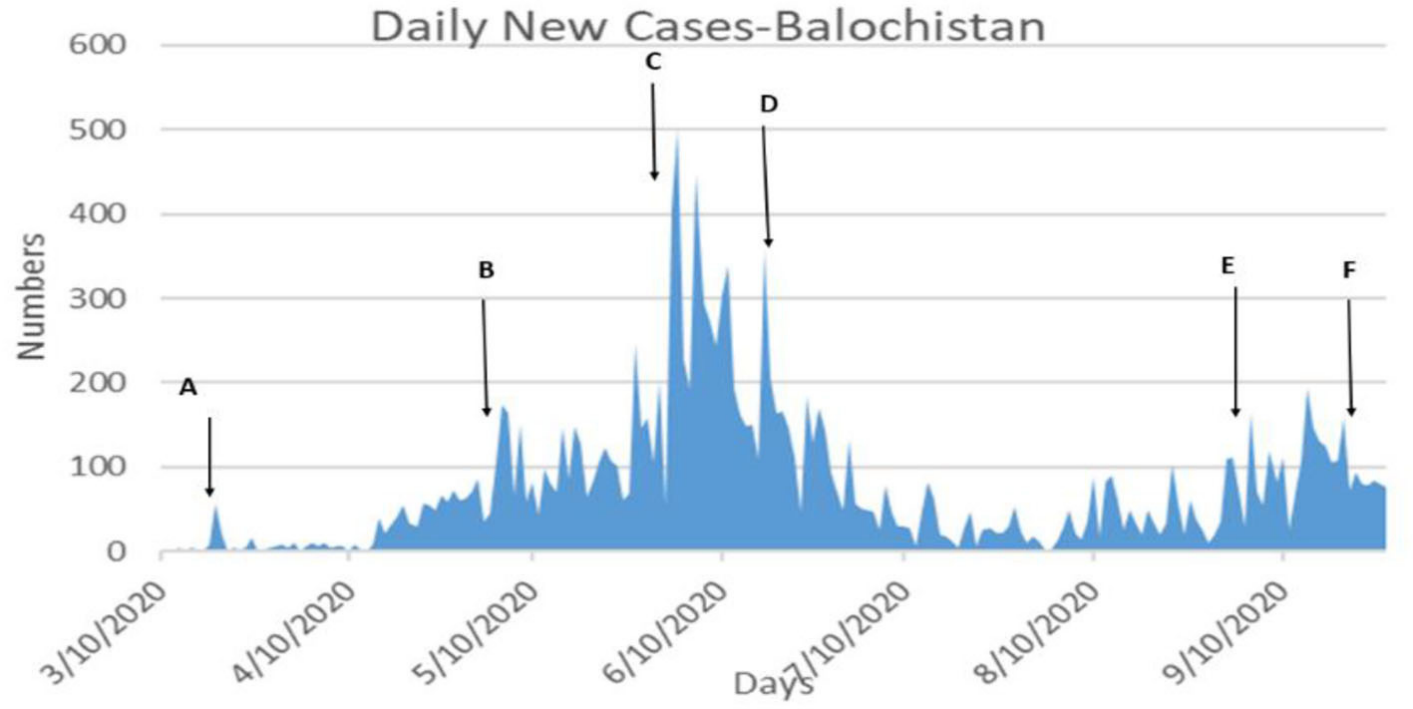
**(A–F)** The number of cases in Baluchistan.

Table 6 shows that the weighted error is the smallest when the conversion rate of an exposed person becomes an infected person, α = 1/4, which is 3.67 and has the best effect. As time increases, the larger the γ, the better the prediction effect. With the development of the epidemic, the cure rate is growing, so as time increases, the prediction effect with a larger γ value will be better. In the actual prediction process, the value of γ can be increased every 6 days on the additional test set (for example, from 1/20, 1/18, and 1/14 until close to 1). On March 20, there was a significant increase in sickness transmission. Between March and September 2020, the number of confirmed cases and deaths in Punjab, Baluchistan, Sindh, KPK, and AJK is shown in Figures 2–6. According to new data, the transmission of the illness has dramatically changed since March. Validate the model parameter through a comparison method (i.e., RMSE, MAE). The result of confirmed cases in the log10 base vs. the last 7 days of training data. The reason for using the last 7 days of training data is because it shows major growth in confirmed cases, as shown in Figure 6.

**Figure 7:**
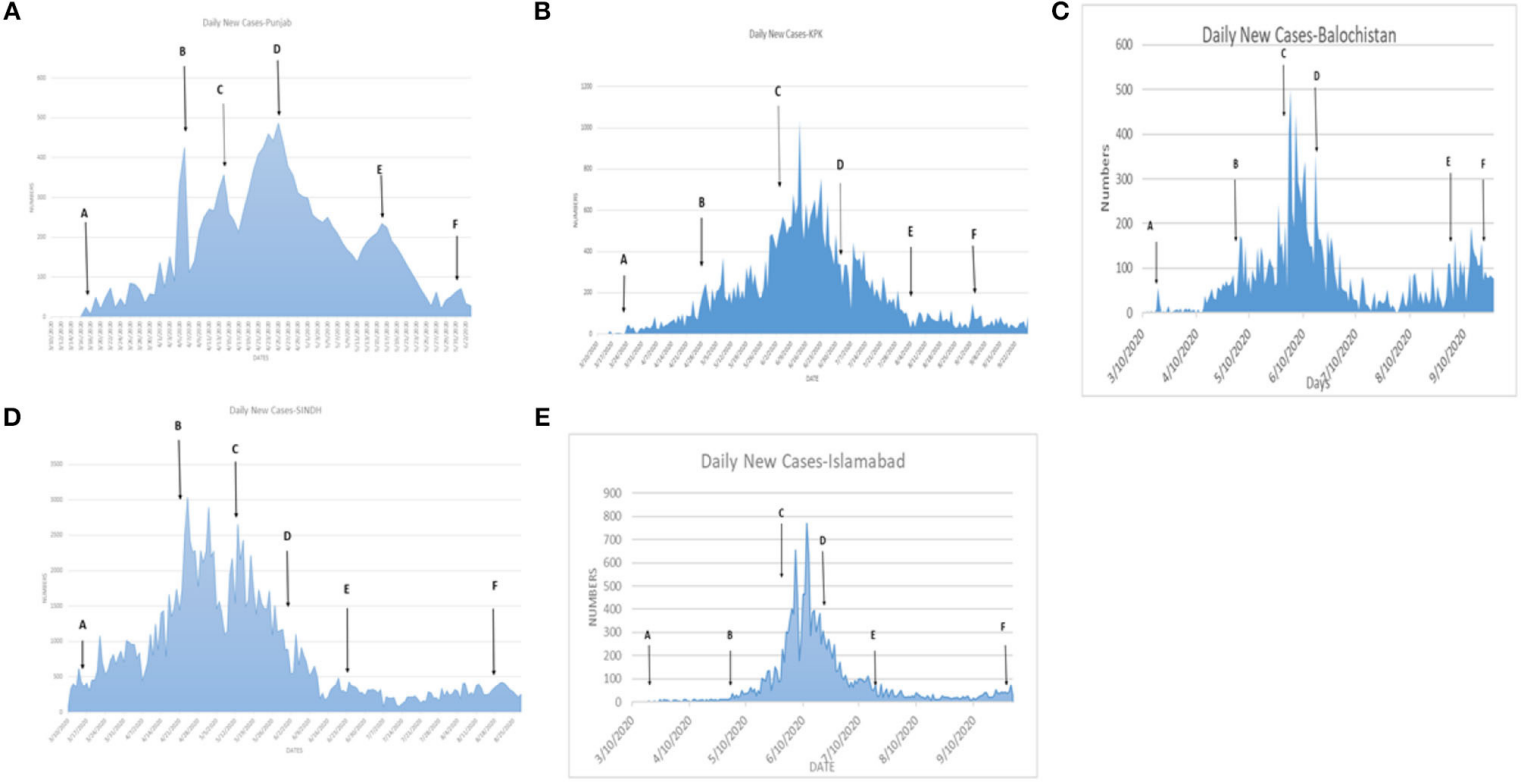
**(A–E)** The number of cases in all provinces of Pakistan.

**Figure 8:**
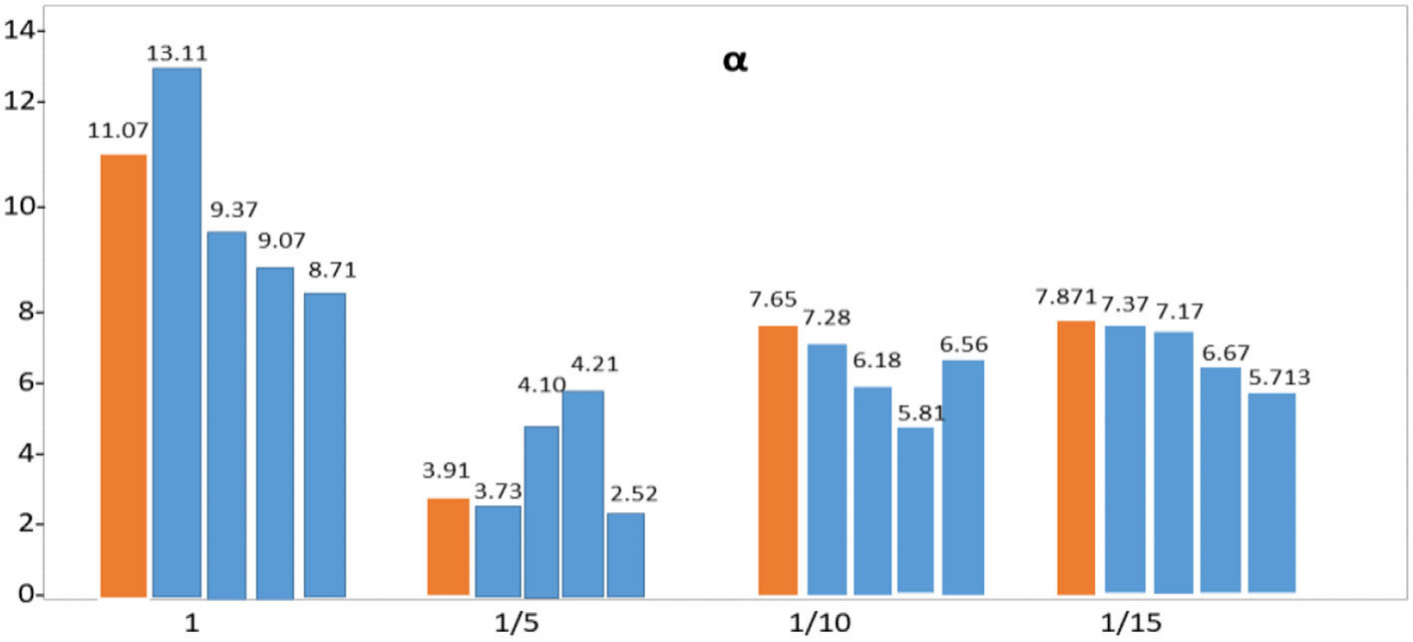
Tuning of SIER model parameter.

Figures 7A–E depicts the most recent projections for each area of Pakistan for the second half of December 2020. Predicted numbers of deaths, recovered cases, and confirmed cases all agree perfectly with the data. They match the information perfectly during the first 6 days, but by the end of the following week, they are more likely to underestimate or misunderstand the observed attributes. This framework's potential to achieve astonishing multiweek expectation levels is demonstrated mathematically in the results to deal with the pandemic's current state and projecting out to future times is shown in Figure 8.

Figure 8 depicts the anticipated outcome based on the use of two models. By adjusting the SEIR model's coefficients repeatedly, the repeated organization helps clarify the framework's evolution and possible modifications.

COVID-19 is contagious and the rate at which the illness spreads from one infected person to another is 2.67; the study needs to know the maximum rate at which the illness may spread. The study used 7-day preparatory information in all models because there is no big trend in Pakistan before walk 2020. SEIR model boundary distinguishes changes in the pandemic development and adjusts the SEIR model coefficient and results to remedy the expectation. The forecast bend, MAE, and RMSE showed that the proposed model in the research is superior to the SEIR model in the investigation. It is possible to conduct this review in different countries, allowing the model to be used in different countries.

**Figure 9:**
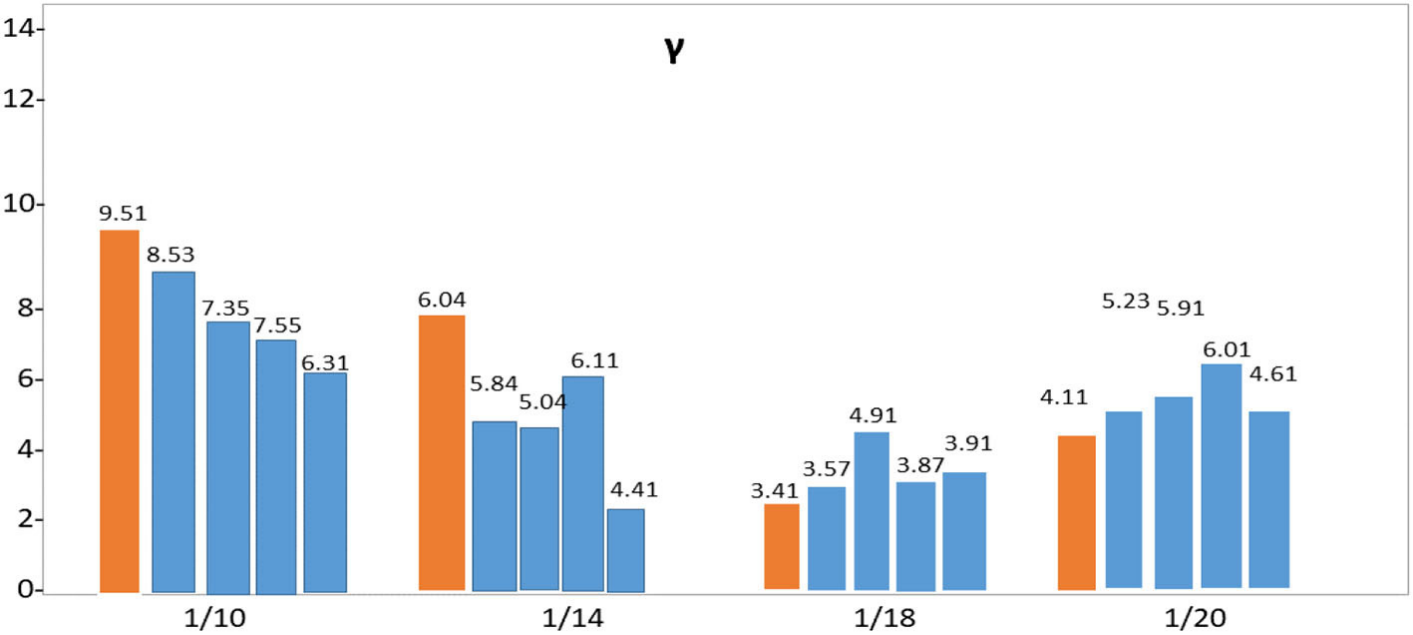
Percentage variation of SIER model parameter.

Although it does not change drastically over time, the SEIR model boundary benefits from several intervening strategies, as seen in Figure 9. Similarly, the SEIR model relies on the number of vulnerable people in the population, and a day-by-day estimate of the framework's relevance for making immediate forecasts began on January 10, followed by estimates on January 14, January 18, and January 20. The osmosis cycle's evaluated status and bounds guide these hypotheses.

**Figure 10:**
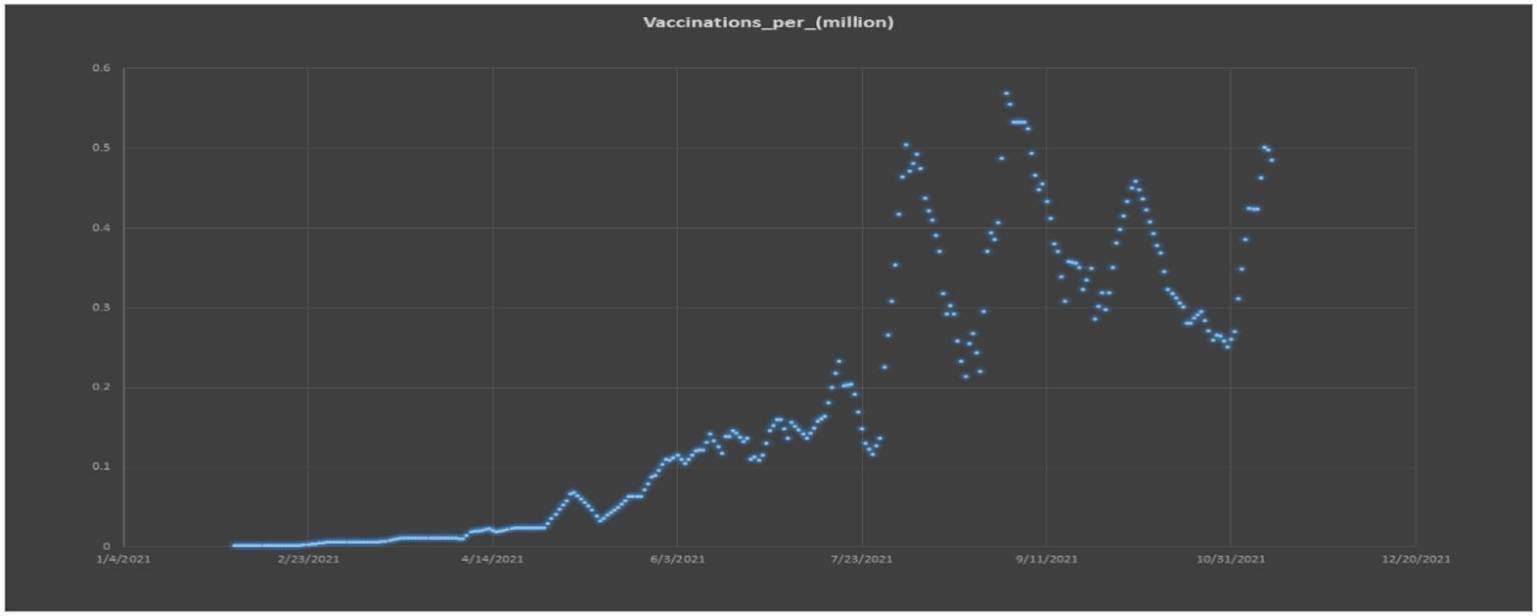
Graphical presentation of the vaccination per million.

Figure 9 also aids in comprehending the Pakistani government's plans for dealing with COVID-19 in each of the country's regions. A time-series graph depicts COVID-19 inoculation in Figure 10, it becomes more apparent that there is a positive vertical pattern in the information. People who provide basic forms of assistance, such as specialists and healthcare workers, need to have their safety and wellbeing protected by nationally recognized clinical standards. People's indifference and gatherings could lead to a significant increase in the number of cases in the future. The public authority must be even more vigilant and implement harsher measures to avoid a climax. In addition, clinical office layouts across the country must be compelled to be modernized.

**Figure 11:**
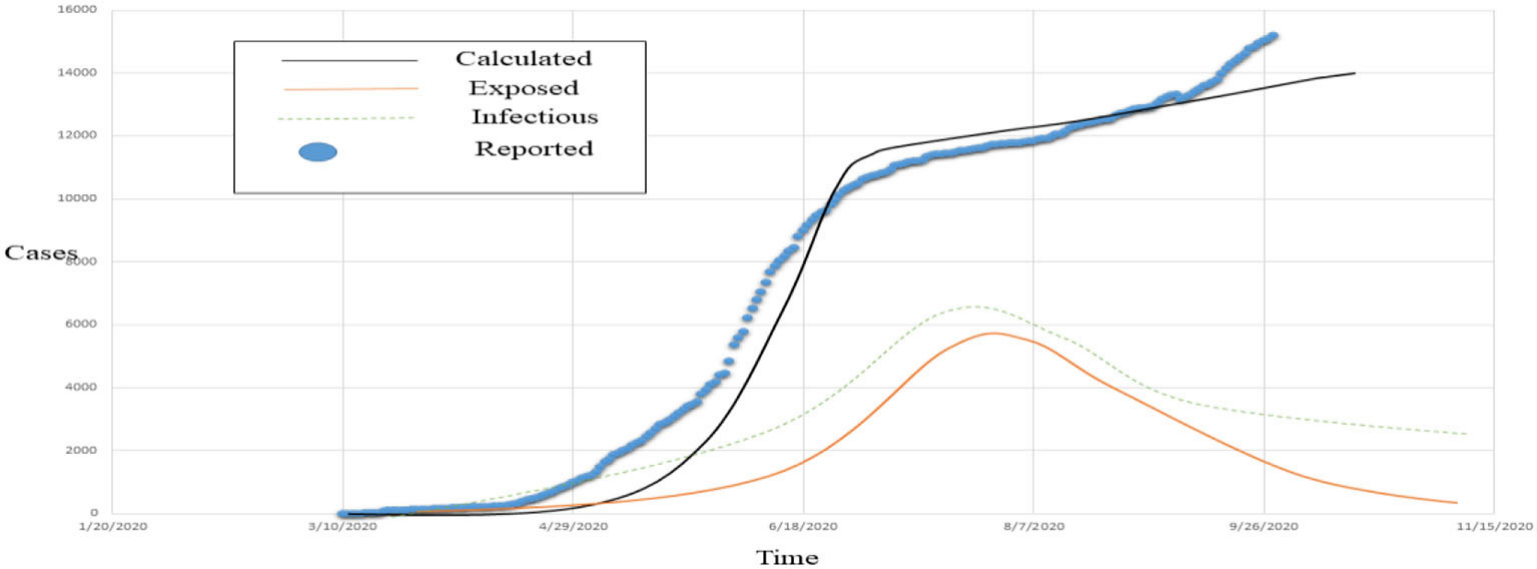
Visualization of observed vs. estimated COVID-19 cases.

**Figure 12:**
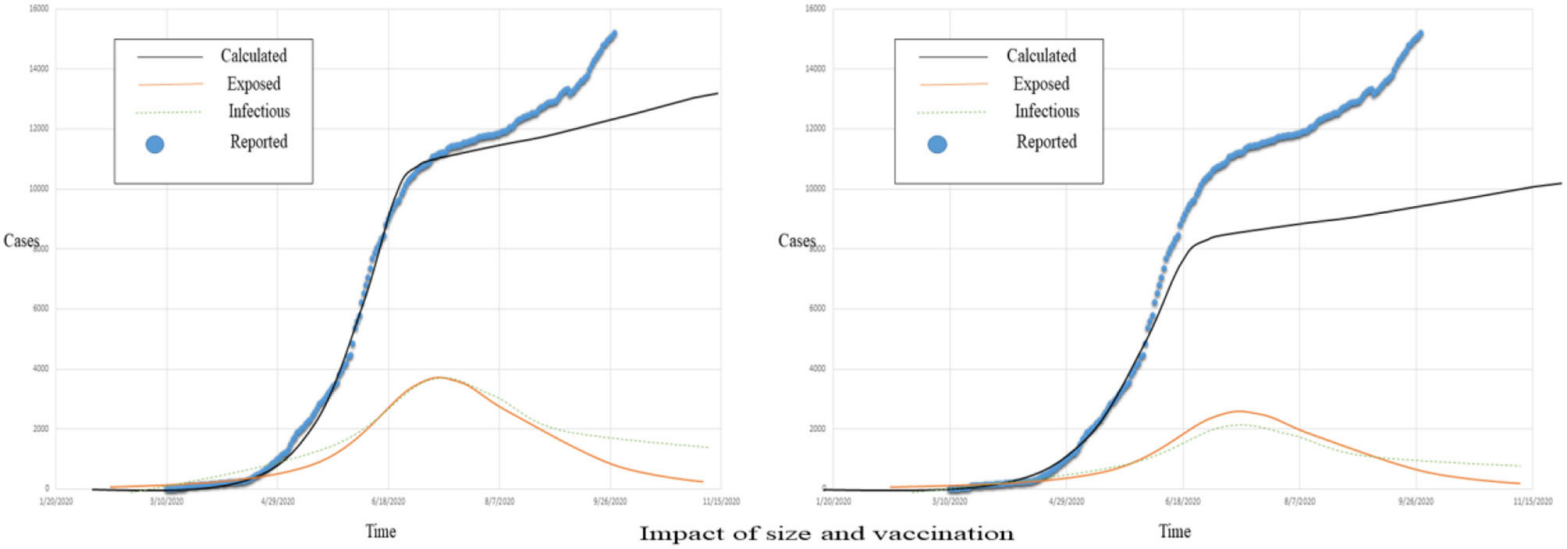
The population size impact on COVID-19 pandemic spread rate.

Figure 11 represents the actual reported, exposed, infectious, and the estimated COVID-19 cases; Figure 12 left graphs show the size, and the right graphs show the vaccination graphs.

Vaccination is proven effective in the stated case, and with the passage of time and awareness, the volume of vaccination is increasing.

## Discussions

In this study, the SEIR model is used to assess COVID-19's effects in Pakistan's Punjab and Sindh provinces and the rest of the country. Compared to other places, the situation in these two towns is dire, and the government of Sindh should take specific measures to protect residents from the spread of COVID-19. Because it considers the interactions between small groups of people spread across Pakistan's many regions, the SEIR model generates new aspects and variables. Others may add classes to the model and update the results if unexpected arrangement executions, such as lockdowns, clever lockdowns, and travel boycotts between cities, are carried out. Even under different models, the advantages can still be quite subtle and arbitrary.

The pandemic model predicts that more people will contaminate than in the days leading up to and following the outbreak. Additional precise data on boundaries will be obtained after the end of infection and may use entire information to anticipate the improvement of (and *R*_0_) with time. Social and familial seclusion are effective strategies, according to these findings. There is no way to prepare for a pandemic in a medical care system that is not designed to handle normal scenarios. As the number of people exposed to pollution grows, illness outbreaks can occur within days. The number of setbacks might be significant if the spread and dominance of illnesses are widespread. One additional lockdown is achieved with a certain space intelligent arrangement where the dispersion is much wider, and it may have a significant impact on preventing this calamity. After a few days of lockdown, educational facilities are allowed to reopen under the authority of approved standard operating procedures. Cooperatives in high-risk areas have been protected, and the public authority has provided help for everyday use goods such as food and water.

## Conclusions

COVID-19 epidemic is simulated and predicted using an enhanced SEIR model with seven phases of infection, including vaccination. The number of deaths, the number of recovered cases, and the number of ongoing cases are analyzed using genuine data. Two weeks into the future, the revised status and parameters are utilized to make short-term projections of the COVID-19 pandemic to assess the system's applicability. The final analysis focused on vaccination's role in disease transmission. As a final step, we hope that our findings will assist policymakers to design psychological treatments that will lessen the psychosocial effects of COVID-19 while also benefiting the most disadvantaged populations that are more likely to suffer from poor health as a result of this pandemic. Based on the validation findings, we can infer that the suggested hybrid model has a reasonable capacity to forecast and decent performance. Long-term projections of the outbreak's dynamics are particularly valuable for healthcare personnel and government authorities. Logistic models cannot predict the number of confirmed diagnoses, but the hybrid model in the research does. The SEIR model can forecast the number of confirmed diagnoses, but it takes a huge quantity of data and repeated revisions to be modified. Experiment results show that the suggested model in this study is superior to the SEIR alone, as demonstrated by the prediction curve, MAE and RMSE values.

### Limitations of SIR and SIER Models

A fresh direction and new way of thinking have been supplied for the COVID-19 pandemic due to the growth in vast amounts of data and the development of computerized reasoning. The premise of artificial intelligence considers the links between objects rather than the substance of the causal relationship. Because we did not want to keep making conclusions based on existing examples of data, we combined the SIR and SEIR restrictions. When the two methods and enhancements are combined, amazing outputs and unmatched concepts result. The creamer model overwhelms the fundamental model's inability to foresee the amount of validated examination. It must incorporate a considerable volume of data into SEIR's model, which can already predict the number of examinations conducted.

### Future Suggestions

These results may also apply to other countries with similar socioeconomic profiles as COVID-19 affects all the countries, and social distancing measures are more or less similar globally. Furthermore, given the recent increase in COVID-19 cases in Pakistan, there is a risk of compliance with procedures for preventative precautions. Following these guidelines and disseminating the right information requires complex awareness campaigns and educational interventions that focus on safe health practices and proper evidence-based information about this disease.

## Data Availability Statement

The original contributions presented in the study are included in the article/supplementary material, further inquiries can be directed to the corresponding authors.

## Author Contributions

WZ: conceptualizing, writing, drafting, and data and methodology. YS: conceptualizing, writing, drafting-original draft, and data and methodology. YL: conceptualizing and writing. WG: data and methodology. All authors contributed to the article and approved the submitted version.

## Funding

The authors thank the financial support from the Natural Science Foundation of China (Grant Number: 71973073).

## Conflict of Interest

The authors declare that the research was conducted in the absence of any commercial or financial relationships that could be construed as a potential conflict of interest.

## Publisher's Note

All claims expressed in this article are solely those of the authors and do not necessarily represent those of their affiliated organizations, or those of the publisher, the editors and the reviewers. Any product that may be evaluated in this article, or claim that may be made by its manufacturer, is not guaranteed or endorsed by the publisher.

## Abbreviations

MSE: Mean squared error
NMSE: Normalized mean squared error
MAE: Mean absolute error
MAPE: Mean absolute percentage error.
